# Ion Uptake in Tall Fescue as Affected by Carbonate, Chloride, and Sulfate Salinity

**DOI:** 10.1371/journal.pone.0091908

**Published:** 2014-03-13

**Authors:** Lei Han, Yang Gao, Deying Li

**Affiliations:** 1 Research Institute of Forestry/Key Laboratory of Forest Cultivation, State Forestry Administration, Beijing, China; 2 Department of Plant Sciences, North Dakota State University, Fargo, North Dakota, United States of America; DOE Pacific Northwest National Laboratory, United States of America

## Abstract

Turfgrass nutrient uptake may be differentially affected by different salts. The objective of this study was to compare nutrient uptake in tall fescue (*Festuca arundinacea* Schreb.) as affected by carbonate, chloride, and sulfate under iso-osmotic, iso-Na^+^ strength conditions. ‘Tar Heel II’ and ‘Wolfpack’ cultivars were subjected to NaCl, Na_2_CO_3_, Na_2_SO_4_, CaCl_2_, NaCl+ CaCl_2_, Na_2_CO_3_+ CaCl_2_, and Na_2_SO_4_+ CaCl_2_, in the range of 0 to 225 mM. There was no cultivar difference regarding K, Na, Mg, and Mn content in shoots. ‘Tar Heel II’ had higher shoot Ca content than ‘Wolfpack’, which were 6.9 and 5.7 g kg^−1^, respectively. In general, K^+^/Na^+^ ratio decreased with increasing salt concentrations, which reached <1 at about 87.5 mM in Na_2_CO_3_ treatment. All salt treatments decreased Mg content in shoot tissues, especially in Na_2_CO_3_ and treatments containing CaCl_2_. Both Ca and Mg content in shoot were higher in the NaCl treatment than the Na_2_SO_4_ and Na_2_CO_3_ treatments. All salt treatments except Na_2_CO_3_ had higher Mn content in shoots compared to the control. In conclusion, nutrient uptake was differently affected by carbonate, chloride, and sulfate which are different in pH, electrical conductivity (EC), and osmotic potential at the same concentration. Adding Ca to the sodium salts increased Ca content and balanced K^+^/Na^+^ in shoots, but did not increase Mg content, which was below sufficient level. Maintaining Mg content in shoots under salinity stress was recommended. The physiological impact of elevated Mn content in shoot under salinity stress requires further study.

## Introduction

Turfgrass is often exposed to salinity stress in salt-affected soils or when recycled water is used for irrigation [1]. Soluble salts in soils exist in various proportions of the cations sodium, calcium, and magnesium, and anions chloride and sulfate. Appreciable amounts of carbonates can be present at pH 9.5 or higher [2]. Excessive salts can be toxic to the grass as well as causing nutrient imbalance and deficiency [3]. As a result, salinity stress often causes poor quality or death of turfgrasses [4]. In addition to using salt tolerant species and cultivars, turfgrass managers need to reduce the salt levels and balance the nutrients levels in soils in order to maintain quality turfgrass [5].

Salinity problems are salt specific. Gao et al. [6] reported that under iso-molar concentration, NaCl had the lowest EC and highest osmotic potential, and induced less growth reduction and physiological stress compared to Na_2_CO_3_, Na_2_SO_4_, and CaCl_2_ in tall fescue (*Festuca arundinacea* Schreb.). In a study with pea (*Pisum sativum* L.), Na_2_CO_3_ had the greatest reduction in osmotic pressure of leaves, followed by Na_2_SO_4_ and NaCl, while the shoot and root dry weight reduction was greater with NaCl, followed by Na_2_CO_3_ and Na_2_SO_4_ [7].

Extensive research has been conducted on warm-season grasses regarding the uptake of ions as affected by salinity stress. Using sea salt mixture in the range of 1 to 42.6 dS m^−1^, Dudeck and Peacock [8] found that K, Mg, Ca, Na content were differentially affected while Mn and Fe remained unchanged with increased salinity in zoysiagrass (*Zoysia* spp. Willd.), seashore paspalum (*Paspalum vaginatum* Swartz), bermudagrass (*Cynodon* spp.), and St. Ausustinegrass [*Stenotaphrum secundatum* (Walt.) Kuntze]. The uptake of K, Ca, and Mg in the shoots and roots of bermudagrass were reduced by NaCl salt [9]. Salinity also reduced the uptake of K, Ca, and Mg in halophytic seashore paspalum [10]. Hameed and Ashraf [11] reported that salt tolerant bermudagrass ecotypes restricted Na uptake in shoots and increased the uptake of K and Ca in shoots and roots.

Research on cool-season grasses has shown that K content in the tissues decreased with increasing salinity levels in creeping bentgrass [12] and tall wheatgrass (*Agropyron elongatum* (Host) Beauv.) [13]. Wyn Jones et al. [14] suggested a threshold K^+^/Na^+^ ratio of 1 for normal growth of plants under salinity stress. Limited information is available on the uptake of other nutrients as affected by salinity stress in cool-season grasses.

Tall fescue is a cool-season turfgrass with moderate to high tolerance to salinity [15]. It also has a wide range of adaptation to heat, drought, and soil pH (4.7 to 9.0) [16], and therefore a great potential to be used in salt affected soils [17]. The objective of this study was to investigate ion uptakes of tall fescue affected by carbonate, chloride, and sulfate salts of sodium as well as the effect of adding Ca to sodium salts.

## Materials and Methods

### Plant materials

Two tall fescue cultivars, ‘Tar Heel II’ (salt tolerant) and ‘Wolfpack’ (salt sensitive) [18], were seeded in April 2010 to containers measuring 4 cm in diameter and 20 cm deep. The growth medium was washed sand with pH of 7.7 and EC of 0.04 dS m^−1^. The plants were maintained in a greenhouse at 25°C (day)/15 °C (night), with a 14-h photoperiod, and a minimum midday *PAR* of 400 μmol m^−2^ s^−1^ supplemented from metal halide lamps. Initially, the plants were watered with distilled water twice a day. Upon germination, the seedlings were watered with half strength Hoagland solution [19] at 10 mL per container twice a week until the 3-leaf stage.

### Experimental design and treatments

**Figure 1:**
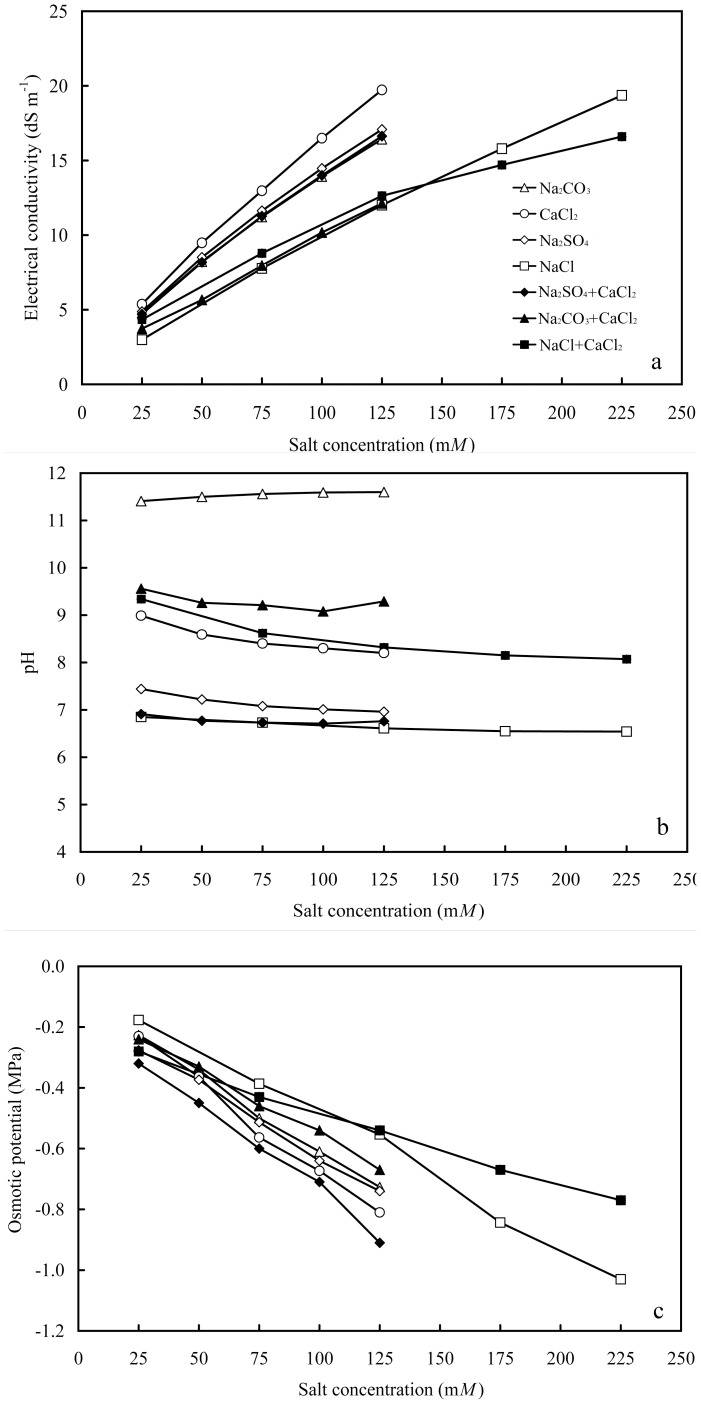
Salt properties at different molar concentrations in deionized water. a) Electrical conductivity, b) pH, and c) osmotic potential.

The seedlings were thinned to three plants of uniform size/stage in each container at the 2-leaf stage and the experimental treatments were initiated at the 4-leaf stage. Seven salt treatments, NaCl, Na_2_CO_3_, Na_2_SO_4_, CaCl_2_, NaCl + CaCl_2_, Na_2_CO_3 +_ CaCl_2_, and Na_2_SO_4_ + CaCl_2_ were used in the study. The concentrations of NaCl and NaCl + CaCl_2_ were at 0, 25, 75, 125, 175, 225 mM, while other salts were at 0, 25, 50, 75, 100, 125 mM. Treatments that had two salts were mixed in 1∶1 ratio. The different concentration range for NaCl was used in order to achieve either a similar range of electric conductivity (EC) or osmotic potential among the four salts (Fig. 1). The osmotic potential was measured with a WP4 dewpoint potential meter (Decagon Devices, Inc. Pullman, WA). The salt treatments were applied to the containers along with full strength Hoagland solution once a week at 20 mL per container, which had pH 6.07, EC 1.3 dS m^−1^, and osmotic potential −0.12 MPa. The EC was measured with an EC meter (model 1054, VWR Scientific, Radnor, PA), and pH was measured using a pH meter (Model 420, Thermal Orion, Pittsburg, PA). The plants were watered with salt solutions every two days to maintain the soil moisture of each container at field capacity based on the evapotranspiration (ET) as determined by weight loss after the previous watering. Over time, the amounts of water changed. However, within one application, all treatments received same amount of water due to very small variations in ET among pots. The experiment was a split-split plot design with three replicates. Cultivar was the main plot factor; salt type was the sub-plot; and salt concentration was the sub-sub plot. Each treatment included 60 plants in 20 containers.

### Tissue and soil analysis

At the end of the 12-week experiment, the shoot biomass was harvested by clipping at the soil surface. The roots were harvested by washing the sand off on a 2-mm sieve with tap water and rinsed with deionized water. The root and shoot dry mass were recorded after drying at 68°C for 48 h. The dry tissues were ground to pass 0.178 mm for tissue nutrient analysis. The content of K, Na, Ca, Mg, and Mn in the tissues roots and shoots were analyzed using an AA7000w flame atomic absorption spectrophotometer (Beijing East & West Electronic Company, Beijing China) following dry ashing in a muffle furnace at 490°C for 8 hours and digestion with 5 M aqua regia [20]–[21]. The K^+^/Na^+^ ratio and the salt concentrations at which this ratio reached the threshold of 1 was derived from the regression lines. The ratio of Ca^2+^ and Mg^2+^ also was calculated for the shoot and root tissues to determine the nutrient balance as affected by different salts.

At the end of study, growth medium pH was determined in a 1∶2 medium: water suspension [22], and the EC was determined in a 1∶5 medium: water extracts [23].

### Statistical analysis

The data were subjected to analysis of variance (ANOVA) using general linear model procedures in SAS 9.2 [24]. Treatment means were separated using Fisher protected least significant difference (LSD) at 0.05 probability level. The PROC REG procedure with a quadratic polynomial model [8], [12] was used for salt content in tissues in response to salt concentrations in the growth medium. Model fitting was evaluated using studentized residual, residual distribution, and Cook's distance.

## Results

### Chemical properties of the growth medium

Seven salt solutions had a range of comparable EC from 3 to 20 dS m^−1^. At the same molar concentration, CaCl_2_ had the highest EC and NaCl had the lowest EC. Na_2_CO_3_ and Na_2_SO_4_ had similar EC because both are sodium salts of divalent anions.

The pH values of these salt solutions were 6.7 to 11.5, with the highest in Na_2_CO_3_ and lowest in NaCl and Na_2_SO_4_+CaCl_2_ (Fig. 1). Adding CaCl_2_ slightly decreased the pH of Na_2_SO_4_, but greatly decreased the pH of Na_2_CO_3_ and increased pH of NaCl. Also, pH was not significantly affected by salt concentrations.

The osmotic potential ranged from −0.2 to −1.05 MPa. NaCl had higher osmotic potential than the other salts. Na_2_CO_3_, Na_2_SO_4_, and CaCl_2_ had a similar osmotic potential at the same molar concentration because of similar anion to cation or cation to anion ratios. Adding CaCl_2_ lowered the osmotic potential of Na_2_SO_4_ as well as that of NaCl at the concentrations below 125 mM (Fig. 1).

At the end of experiment, soil tests showed that the soil EC followed a linear trend from 0.2 to 0.45 dS m^−1^ in relation to the concentrations of salt solutions in the treatments except for Na_2_SO_4_+CaCl_2_, which had highest EC ranging from 0.32 to 0.93 dS m^−1^ (data not shown). Also, NaCl, Na_2_SO_4_, Na_2_CO_3_, NaCl+CaCl_2_ and Na_2_CO_3_+CaCl_2_ increased the pH of the growth medium by 1.5, 0.9, 3.0, 1.0, and 2.5 units from the original value of 7.7, respectively; whereas CaCl_2_ and Na_2_SO_4_+CaCl_2_ treatments did not change the pH of the growth medium (data not shown).

### K^+^/Na^+^ ratio in shoots

**Figure 2:**
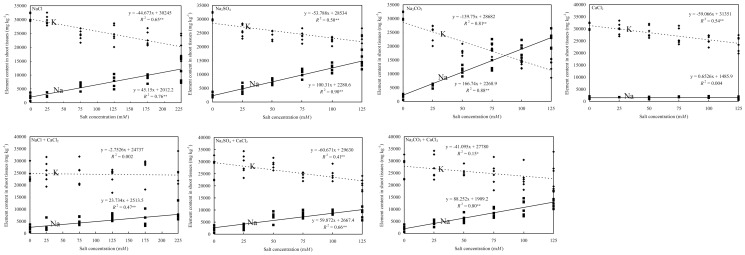
K and Na content in shoot tissues of tall fescue as affected by sodium chloride, sodium carbonate, sodium sulfate, and calcium chloride. Ion content was measured in weekly intervals over a period of five weeks. Data were combined for two cultivars, Tar Heel II and Wolfpack. * and ** denote significance at 0.05 and 0.01 probability levels, respectively.

There was no difference between the two cultivars tested regarding K and Na content in shoot tissues. Shoot tissue K content was affected by salt types, salt concentrations, and their interactions (Table 1), and it was the lowest in Na_2_CO_3_ treatment followed by NaCl and Na_2_SO_4_ treatments (Table 2). Shoot Na content was the highest in the Na_2_CO_3_ treatment, followed by Na_2_SO_4_, Na_2_CO_3_+CaCl_2_, Na_2_SO_4_+CaCl_2_, and NaCl (Table 2). As a result, the Na_2_CO_3_ treatment had the lowest K^+^/Na^+^ ratio of 1.76 (ranging from 4.57 to 0.59), Na_2_SO_4_ treatment had a K^+^/Na^+^ ratio of 3.07 (ranging from 5.95 to 1.63), and NaCl treatment had a K^+^/Na^+^ ratio of 4.82 (ranging from 11.95 to 1.92). In general, K^+^/Na^+^ ratio decreased with the increase of salt concentrations (Fig. 2). Although CaCl_2_ treatment alone decreased shoot K content, adding CaCl_2_ to other salts increased K^+^/Na^+^ ratios in shoots because of the Na uptake was reduced by CaCl_2_ (Fig. 2). The K^+^/Na^+^ ratio in shoot tissues fell below one at about 87.5 mM in the Na_2_CO_3_ treatment. However, K^+^/Na^+^ ratio in shoot tissues was greater than one in the Na_2_CO_3_+CaCl_2_ treatment and other salt treatments (Fig. 2). The K^+^/Na^+^ ratio in shoot of NaCl+CaCl_2_, Na_2_SO_4_+CaCl_2_, and Na_2_CO_3_+CaCl_2_ treatments was 6.03 (12.4 to 2.96), 4.79 (11.5 to 2.45), and 3.75 (6.92 to 1.82), respectively (Fig. 2). K^+^/Na^+^ ratio was the lowest in Na_2_CO_3_ treatment indicating that osmotic potential as well as pH may be factors in addition to the Na^+^ effect.

**Table 1:** Analysis of variation of ion uptake in the shoots of tall fescue as affected by cultivar, salt types and salt concentrations.

Source of variation	df	K	Na	Ca	Mg	Mn
	***P***					
Cultivar (V)	1	0.094	0.051	0.04	0.057	0.021
Salt type (S)	6	<0.0001	<0.0001	<0.0001	<0.0001	<0.0001
Concentration (C)	5	<0.0001	<0.0001	<0.0001	<0.0001	<0.0001
V*S	6	0.157	<0.0001	<0.0001	0.003	0.0002
V*C	5	0.190	<0.0001	0.149	0.435	0.0035
S*C	30	<0.0001	<0.0001	<0.0001	<0.0001	<0.0001
V*S*C	30	0.009	<0.0001	0.051	0.071	<0.0001

**Table 2:** Ion content in tall fescue as affected by different salts with data pooled for cultivars (Tar Heel II and Wolfpack) and salt concentrations.

	K		Na		Ca		Mg		Mn	
Salt types	Shoot	Root	Shoot	Root	Shoot	Root	Shoot	Root	Shoot	Root
	**g kg^−1^**								**mg kg^−1^**	
NaCl	25.50c^a^	5.44d	6.71d	12.56a	3.58f	8.57f	1.56b	1.22d	99b	38c
Na_2_SO_4_	25.38c	3.52e	8.55b	7.49c	3.09g	8.28f	1.48c	1.54b	82d	32e
Na_2_CO_3_	20.25d	2.56f	12.69a	4.33f	2.87g	12.51d	1.08g	1.70a	54f	34d
CaCl_2_	27.62b	9.65a	1.53g	1.59g	11.29a	17.07b	1.22f	1.36c	107a	40b
NaCl+CaCl_2_	25.30c	6.61c	4.99e	6.70d	8.57b	13.19d	1.28e	1.48c	107a	37c
Na_2_SO_4_+CaCl_2_	26.04c	5.60d	6.41d	8.30b	6.76d	16.02c	1.29e	1.37c	102b	38c
Na_2_CO_3_+CaCl_2_	25.71c	3.18e	7.42c	4.95e	7.77c	20.31a	1.42d	1.38c	90c	46a
Control	31.10a	8.23b	2.15f	1.66g	4.59e	11.37e	1.74a	1.56b	70e	24f

a means followed by same letter within a column are not different at 0.05 probability level.

### K^+^/Na^+^ ratio in roots

**Figure 3:**
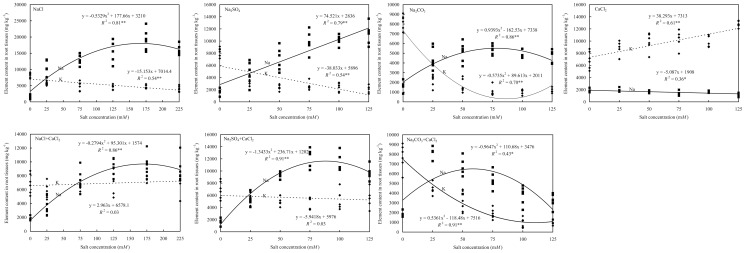
K and Na content in root tissues of tall fescue as affected by sodium chloride, sodium carbonate, sodium sulfate, and calcium chloride. Ion content was measured in weekly intervals over a period of five weeks. Data were combined for two cultivars, Tar Heel II and Wolfpack. * and ** denote significance at 0.05 and 0.01 probability levels, respectively.

There was no difference between the two cultivars tested in root K and Na content (Table 3). The K and Na content in root tissues were affected by salt types, concentration, and the interaction between salt type and concentration (Table 3). The CaCl_2_ treatment increased root K content compared to the control, while other salts decreased K content in root tissues with Na_2_CO_3_ treatment causing the greatest reduction (Table 2). The K content in root tissues decreased with increasing concentrations of NaCl, Na_2_SO_4_ and Na_2_CO_3_ but increased with increasing concentrations of CaCl_2_ (Fig. 3). The K content in root tissues decreased in quadratic fashion with Na_2_CO_3_ concentration and in linear with Na_2_SO_4_ and NaCl. The slope of the regression was −38.0 mg kg^−1^ mM^−1^ in Na_2_SO_4_, steeper in than −15.2 mg kg^−1^ mM^−1^ in NaCl (Fig. 3). Adding CaCl_2_ to NaCl, Na_2_SO_4_ and Na_2_CO_3_ resulted in a lower rate of decrease in root K content (Fig. 3). NaCl treatment had a higher Na content in root tissues than Na_2_SO_4_ and Na_2_CO_3_. The root Na content in CaCl_2_ treatment was at the same level as in the control (Table 2). The Na content in root tissues decreased with the increasing concentration of CaCl_2_ treatment (Fig. 3). Adding CaCl_2_ to NaCl did not change the increasing rate of Na in roots (Fig. 3). However, adding CaCl_2_ to Na_2_SO_4_ and Na_2_CO_3_ at above 75 mM decreased the rate of Na uptaking rate in roots (Fig. 3). The K^+^/Na^+^ ratio in roots reached <1 in all salt treatments except CaCl_2_. The threshold of K^+^/Na^+^ <1 was reached at 10 mM in NaCl treatment, and at 100 mM in NaCl+CaCl_2_ treatment. However, K^+^/Na^+^ ratio exhibited <1 in Na_2_SO_4_ and Na_2_CO_3_ treatments at 25 mM with or without CaCl_2_ (Fig. 3).

**Table 3:** Analysis of variation of ion content in the roots of tall fescue as affected by cultivar, salt types and salt concentrations.

Source of variation	df	K	Na	Ca	Mg	Mn
		***P***				
Cultivar (V)	1	0.05	0.436	0.218	0.085	0.800
Salt type (S)	6	<0.0001	<0.0001	<0.0001	<0.0001	<0.0001
Concentration (C)	5	<0.0001	<0.0001	<0.0001	<0.0001	<0.0001
V*S	6	<0.0001	0.144	0.244	<0.0001	<0.0001
V*C	5	0.371	0.397	0.037	<0.0001	<0.0001
S*C	30	<0.0001	<0.0001	<0.0001	<0.0001	<0.0001
V*S*C	30	<0.0001	<0.0001	0.528	<0.0001	<0.0001

### Ca^2+^ and Mg^2+^ ratio in shoots

Both Ca and Mg content in shoots were differently affected by salt type, salt concentration, and the interaction between the two factors (Table 1). There were no differences between two cultivars regarding the Mg content in shoots. However, ‘Tar Heel II’ had a higher shoot Ca content of 6.9 g kg^−1^ than ‘Wolfpack' of 5.7 g kg^−1^. The higher Ca content may have been involved in the mechanism of salinity tolerance to balance the ions as reported elsewhere [10]–[11]. All three sodium salt treatments decreased Ca content in the shoots, whereas CaCl_2_ treatment increased Ca content compared to the untreated control (Table 2). Shoot Ca content decreased with the increasing concentrations of NaCl, Na_2_SO_4_ and Na_2_CO_3_, but increased with the increasing concentrations of CaCl_2_ (Table 4). Adding CaCl_2_ to NaCl, Na_2_SO_4_ and Na_2_CO_3_ resulted in increases of Ca content in shoots (Table 4). All salt treatments decreased Mg content in shoot tissues, especially Na_2_CO_3_ and the treatments containing CaCl_2_ as compared to the untreated control. Both Ca and Mg content in shoot were higher in the NaCl treatment than the Na_2_SO_4_ and Na_2_CO_3_ treatments (Table 2). Shoot Ca^2+^/Mg^2+^ ratio decreased with increasing concentrations of NaCl and Na_2_SO_4_, which were from 2.6 to 2.0 and 2.2 to 1.3, respectively (Table 4). The Ca^2+^/Mg^2+^ ratios increased with the increasing concentrations of Na_2_CO_3_ and CaCl_2_, which were from 2.7 to 2.9 and 1.9 to 14.8, respectively (Table 4). Adding CaCl_2_ increased Ca^2+^/Mg^2+^ ratios in the shoot tissues, which were from 2.2 to 11.5, 2.2 to 8.0, and 2.1 to 10.6, for NaCl+CaCl_2_, Na_2_SO_4_+CaCl_2_, Na_2_CO_3_+CaCl_2_, respectively (Table 4).

**Table 4:** Ion uptake in tall fescue as affected by different salts with data pooled for cultivars (Tar Heel II and Wolfpack) and salt concentrations.

	Ca		Mg		Mn	
Salt types	Regression^a^	*R* ^2^	Regression	*R* ^2^	Regression	*R* ^2^
	**Shoot**					
NaCl	Y = −7.4x+4347	0.56**	Y = −1.2x+1680	0.21*	Y = 0.2x+78	0.54**
Na_2_SO_4_	Y = −0.07x+3830	0.66**	Y = −0.07x+1738	0.52**	Y = 0.15x+74	0.31*
Na_2_CO_3_	Y = −16.3x+3883	0.50**	Y = −8.59x+1613	0.83**	Y = −0.31x+74	0.81**
CaCl_2_	Y = −1.21×^2^+243.16x+3026	0.86**	Y = −3.70x+1455	0.51**	Y = −0.007×^2^+1.13x+76	0.70**
NaCl+CaCl_2_	Y = 32.02x+4627	0.67**	Y = −3.35x+1632	0.59**	Y = −0.001×^2^+0.39x+84	0.51**
Na_2_SO_4_+CaCl_2_	Y = 37.33x+4423	0.56**	Y = −5.07x+1608	0.51**	Y = −0.005×^2^+0.94x+74	0.59**
Na_2_CO_3_+CaCl_2_	Y = 66.3x+3627	0.78**	Y = −5.49x+1762	0.59**	Y = 0.006×^2^+0.87x+70	0.52**
	Root					
NaCl	Y = −26.77x+11355	0.78**	Y = −2.93x+1524	0.37*	Y = 0.096x+28	0.67**
Na_2_SO_4_	Y = −59.13x+11978	0.82**	Y = −4.42x+1811	0.20*	Y = 0.109x+25	0.50**
Na_2_CO_3_	Y = 14.91x+11578	0.16*	Y = 4.94x+1393	0.37*	Y = 0.128x+26	0.63**
CaCl_2_	Y = 58.63x+13401	0.46**	Y = −4.05x+1616	0.46**	Y = 0.076x+29	0.50**
NaCl+CaCl_2_	Y = −0.31×^2^+70.9x+11602	0.39*	Y = −1.83x+1673	0.16	Y = 0.146x+31	0.65**
Na_2_SO_4_+CaCl_2_	Y = −1.64×^2^+228.9x+11103	0.63**	Y = 4.13x+1109	0.08	Y = 0.203x+26	0.52**
Na_2_CO_3_+CaCl_2_	Y = 163.2x+10112	0.91**	Y = −11.14x+2072	0.46**	Y = 0.34x+23	0.78**

a Y (g kg^−1^) as a response variable affected by salt concentration × (mM).

*, **, *** significant at 0.05, 0.01, and 0.001 probability levels, respectively.

### Ca^2+^ and Mg^2+^ ratio in roots

Both Ca and Mg content in root tissues were affected by salt type, salt concentration, and the interaction between the two factors. No differences were detected between the two cultivars (Table 3). Only NaCl and Na_2_SO_4_ treatments had lower Ca content in roots than the control (Table 2). Root Ca content decreased with the increasing concentrations of NaCl and Na_2_SO_4_, and increased with the increasing concentrations of Na_2_CO_3_ and CaCl_2_ (Table 2). Adding CaCl_2_ to NaCl, Na_2_SO_4_ and Na_2_CO_3_ resulted in a linear increase of Ca content in Na_2_CO_3_+CaCl_2_ treatment and quadratic increase of Ca in NaCl+CaCl_2_ and Na_2_SO_4_+CaCl_2_ treatments (Table 4).

The Na_2_CO_3_ treatment showed higher Mg content in roots than the control, while all treatments containing chloride had lower Mg content in root than the control (Table 2). The root Ca^2+^/Mg^2+^ ratio decreased in Na_2_SO_4_ and Na_2_CO_3_ treatments with the increase of salt concentration, from 8.0 to 4.8 and 7.8 to 5.8, respectively (Table 2). The Ca^2+^/Mg^2+^ ratio in root increased with the increasing concentrations of NaCl, CaCl_2_, NaCl+CaCl_2_, Na_2_SO_4_+CaCl_2_ and Na_2_CO_3_+CaCl_2_, which were 7.3 to 7.8, 7.0 to 18.1, 7.0 to 11.8, 7.0 to 8.1, and 6.4 to 37.4, respectively (Table 2).

### Mn content in shoot

The Mn content in shoots showed no difference between two cultivars (Table 1). The Na_2_CO_3_ treatment had lower Mn content in shoots than the control, while other salt treatments all had higher Mn content in shoots (Table 2). The shoot Mn content increased linearly with the increase of salt concentrations in NaCl and Na_2_SO_4_ treatments, and decreased linearly with the increase of Na_2_CO_3_ concentrations (Table 4). Shoot Mn content showed quadratic increase with the increasing concentrations of CaCl_2_, NaCl+CaCl_2_, Na_2_SO_4_+CaCl_2_, and Na_2_CO_3_+CaCl_2_ (Table 4).

### Mn content in root

Root Mn content also was significantly affected by salt treatments without difference between the two cultivars tested (Table 3). All salt treatments had higher Mn content than the control (Table 2). Root Mn content increased linearly significantly with salt concentrations (Table 4).

### Biomass of shoot and root

**Figure 4:**
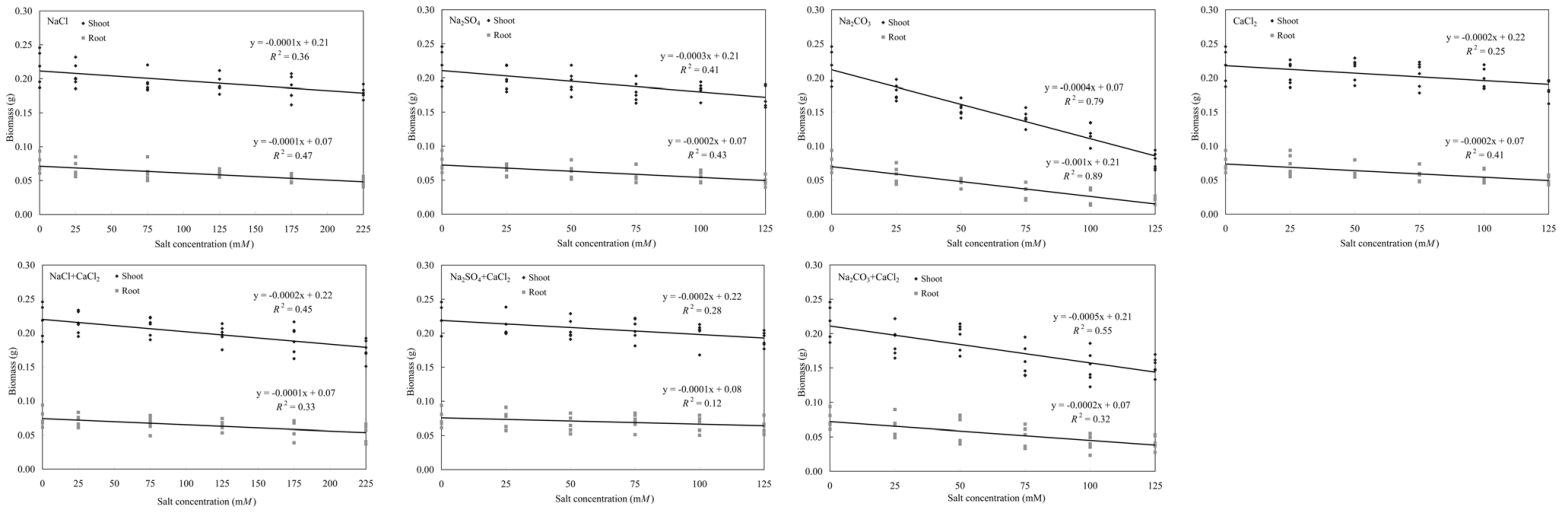
Shoot and root biomass of tall fescue as affected by sodium chloride, sodium carbonate, sodium sulfate, and calcium chloride. Data were combined for two cultivars, Tar Heel II and Wolfpack. * and ** denote significance at 0.05 and 0.01 probability levels, respectively.

Different salts affected differently the shoot and root biomass of tall fescue (Fig. 4). Shoot and root biomass decreased similarly as salt concentration increased for all salts with the most reduction happened in Na_2_CO_3_ treated plants. Adding CaCl_2_ to other salts did not seem to alleviate the biomass reduction except for the Na_2_CO_3_ treated plants. Overall, at a given molar concentration different salts affected tall fescue differently although such difference attributed to pH as well as osmotic potential. Further research is necessary to differentiate the effects of pH and osmotic potential.

## Discussion

As reported previously, salt treatments (with the exception of Na_2_CO_3_) did not cause significant difference in growth [6]. Therefore, the comparison of ion uptake among the treatments should be separated from the ion removal due to mowing or harvest. Since all nutrients were sufficient in the growth medium, any nutrient deficiency as determined by tissue analysis was primarily caused by nutrient imbalance.

Calcium was the only mineral nutrient which showed differences between the two cultivars tested. Although Ca content could affect other nutrients in the shoots, it did not translate into detectable differences between two cultivars in this study. Cultivar differences in Ca and Mg uptake also were reported previously [25]–[26]. Gao et al. [6] reported that ‘Tar Heel II’ and ‘Wolfpack’ were not differentiated in physiological responses to NaCl, Na_2_SO_4_, Na_2_CO_3_, and CaCl_2_ at a wide range of concentrations. The ranking of ‘Tar Heel II’ as more salt tolerant than ‘Wolfpack’ by Wipff and Rose-Fricker was based on the survival rates of mature plants in one unrepeated greenhouse study [18]. Additional study is needed to confirm the difference of salinity tolerance between the two cultivars and the role of tissue Ca content in salinity tolerance.

The content of K in shoot tissue in single salt treatments ranked similar to their pH levels. Shoot K content was adversely affected by Na or Ca in the salt treatments. The K^+^/Na^+^ ratio in roots reached <1 in all sodium salt treatments (Fig. 3). However, because of the active uptake of K, only Na_2_CO_3_ treatment resulted a K^+^/Na^+^ ratio of <1 in shoot tissues. Although the K loading in roots increased with CaCl_2_ concentration, it did not translate into increased K content in the shoot tissues. Despite the decrease of K^+^/Na^+^ ratio, K content in shoot tissues was below 15 g kg^−1^ in the Na_2_CO_3_ treatment only, which is considered the sufficiency level for tall fescue [27].

Both Ca and Mg content in shoot tissues decreased significantly with increasing salt concentrations of NaCl, Na_2_SO_4_ and Na_2_CO_3_. The results of this study agreed with the reports by MacAdam et al. [28] that irrigating with ground water with high levels of sulfates and sodium, but low content of Ca (149.1 to 238 mg L^−1^) and Mg (40.6 to 98 mg L^−1^) resulted in lower Ca uptake and no changes of Mg content in tall fescue leaves as application rate increased. However, when the irrigation water had a higher concentration of Ca (553.7 mg L^−1^) and Mg (169.7 mg L^−1^), the Ca content in the shoots returned to a level found in the control, and Mg content also was increased [28]. In the present study, Ca content in shoot tissue was above the sufficient level of 5 g kg^−1^, but Mg content was below the sufficient range of 1 to 4 g kg^−1^ [29].

Adding Ca in the sodium salts increased Ca content in the shoots but also the ratio of Ca^2+^/Mg^2+^. Therefore, Mg must also be added to maintain the Ca^2+^/Mg^2+^ ratio as well as its sufficiency level. Although carbonate may cause Ca concentration to decrease in soil solution, it did not explain the decreased Ca content in the shoots because the Ca content in the roots was not affected by Na_2_CO_3_ treatment. The lowered shoot Ca content in Na_2_CO_3_ treatment could possibly be caused by the high pH that prohibit transportation of Ca from root to shoot. Simson et al. [30] reported that crop growth and yield was not affected by the Ca^2+^/Mg^2+^ ratio in soil as suggested by earlier research. Although the present study was not designed to compare the effect of Ca^2+^/Mg^2+^ ratios in growth medium on the uptake of nutrients by tall fescue, the results suggested that there was not a clear correlation between the Ca^2+^/Mg^2+^ ratio in shoot tissues and that in the growth medium.

When tall fescue is used as a forage crop, a high Mg content in the shoot tissues is required to avoid grass tetany. An index of K/(Ca+Mg) in terms of molar concentration is commonly used for the assessment of the sufficiency level of Mg, and a ratio of <2.2 is considered sufficient [25]. In the present study, the index was 2.27 to 2.32, 2.29 to 2.68, and 2.28 to 2.51 for NaCl, Na_2_SO_4_ and Na_2_CO_3_ treatments at different concentration levels, respectively. The K/(Ca+Mg) index was below 2.2 in other salt treatments containing CaCl_2_. However, the tissue nutrients were not balanced under salinity stress, and the index may not be adequate for the purpose of assessing Mg sufficiency because the net Mg content was either decreased or unchanged at different salt concentrations in all treatments.

Deficiency of Mn is likely to happen in soils with high sodium content or high pH [27]. However, decrease of Mn happened only in the Na_2_CO_3_ treatment which had a pH of 11.5. Other salt treatments increased Mn content in the shoot tissues with increasing salt concentration. In Na_2_CO_3_ treatment, Mn content in shoot tissue showed a similar trend as Ca content; it decreased with salt concentration despite the increased loading in the root. Since Mn was an unlimited soluble form provided by the Hoagland solution, it could have been taken up along with Na into shoot tissues. Similar results were reported in a study by Chen et al. [31] where dairy manure with low Ca and Mg contents but a high pH (7.37 to 8.19), and a high EC (4.54 to 7.78 dS m^−1^) increased Mn uptake by tall fescue compared to the untreated control and other composts with lower pH, EC, and higher Ca, Mg, and Mn content. Soil modified with flue gas desulfurization products, which contained Ca (509 g kg^−1^), Mg (24.4 g kg^−1^), and Mn (101 mg kg^−1^) and with high salinity (5.58 dS m^−1^) and pH (8.68), resulted in elevated Ca and Mn content and decreased K content in tall fescue leaves compared to the control or CaSO_4_ treatments [32]. In all those cases, the elevated Mn contents in tall fescue leaves were far below the toxic level of 500 mg kg^−1^. Therefore, the physiological function of Mn under salinity stress needs further study.

## Conclusions

Mineral nutrients were differently affected by carbonate, chloride, and sulfate of sodium. Some of the variables were attributed to their differences in pH, some to EC or osmotic potential. Adding Ca to the sodium salts could maintain the Ca content in the shoot and alleviate the imbalance of K and Na. The decrease of Mg content in shoots was not alleviated by adding Ca. Therefore, improving Mg content in shoots may be needed if tall fescue is to be used as forage under salinity stress. Increasing the Mg content in shoots also may be needed because it fell below sufficient levels under salinity stress regardless of the existence of an optimum Ca^2+^/Mg^2+^ ratio. The impact of increased uptake of Mn under salinity stress requires further study.
